# Efficacy of Fixed Low‐Power Long‐Duration Radiofrequency Ablation of Ventricular Arrhythmias Originating From the Left Ventricular Summit

**DOI:** 10.1002/joa3.70339

**Published:** 2026-05-03

**Authors:** Takuro Masuda, Koji Nakagawa, Satoshi Kawada, Tomofumi Mizuno, Akira Ueoka, Saori Asada, Masakazu Miyamoto, Nobuhiro Nishii, Kazufumi Nakamura, Hiroshi Morita, Shinsuke Yuasa

**Affiliations:** ^1^ Department of Cardiovascular Medicine Okayama University Graduate School of Medicine, Dentistry and Pharmaceutical Sciences Okayama Japan; ^2^ Department of Cardiovascular Medicine Kochi Health Sciences Center Kochi Japan; ^3^ Department of Cardiovascular Medicine Fukuyama Cardiovascular Hospital Fukuyama Japan; ^4^ Department of Cardiovascular Medicine Kagawa Prefectural Central Hospital Takamatsu Japan; ^5^ Department of Cardiovascular Therapeutics Okayama University Graduate School of Medicine, Dentistry and Pharmaceutical Sciences Okayama Japan

**Keywords:** left ventricular summit, long‐duration radiofrequency ablation, low power, ventricular arrhythmias

## Abstract

**Background:**

Radiofrequency catheter ablation (RFCA) of ventricular arrhythmias originating from the left ventricular summit (LVS‐VAs) remains challenging.

**Objective:**

The present study aimed to assess the efficacy and safety of endocardial fixed low‐power long‐duration RFCA (LPLD RFCA), compared with conventional RFCA in the treatment of LVS‐VAs.

**Methods:**

We retrospectively analyzed data from 47 patients with LVS‐VAs: 17 underwent LPLD RFCA defined as RF delivery at a fixed power of 20 W or 25 W for 120–180 s, and 30 received conventional RFCA. Procedural outcomes, complications, and follow‐up data were compared.

**Results:**

The LPLD RFCA group had significantly lower mean RF power and a longer total application time compared to the conventional group (20 ± 1.0 W vs. 36 ± 5.4 W, and 30 ± 13 min vs. 15 ± 11 min, respectively; both *p* < 0.01). Acute procedural success was achieved in 15 of the 17 patients (88%) in the LPLD RFCA group and in 17 of the 30 patients (56%) in the conventional RFCA group (*p* < 0.05). No major complications, including steam pops or pericardial effusion, occurred in either group. The long‐term efficacy of the fixed LPLD RFCA was maintained during the 16‐month follow‐up.

**Conclusions:**

Endocardial RFCA using a fixed LPLD RFCA strategy was associated with a higher acute success rate without major complications compared with conventional RFCA in patients with LVS‐VAs. This approach may represent a feasible alternative ablation strategy for LVS‐VAs.

## Graphical abstract

Efficacy and safety of a fixed low‐power, long‐duration radiofrequency catheter ablation of ventricular arrhythmias originating from the left ventricular summit.

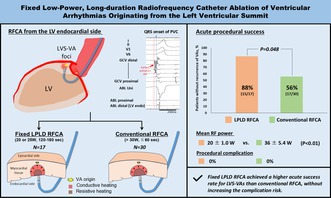

**Abbreviations**
AIV: Anterior interventricular vein
GCV: Great cardiac vein
LCC: Left coronary cusp
LPLD: Low‐power long‐duration
LV: Left ventricle
LVS: Left ventricular summit
RF: Radiofrequency
RFCA: Radiofrequency catheter ablation
RVOT: Right ventricular outflow tract
VA: Ventricular arrhythmia

## 1 Introduction

Radiofrequency (RF) catheter ablation (RFCA) is a well‐established, effective treatment for idiopathic ventricular arrhythmias (VAs) originating from subendocardial sites in both the right and left ventricles [1]. However, treating VAs arising from the epicardium or sub‐epicardium remains challenging due to anatomical barriers, myocardial wall thickness, and limited catheter accessibility. Among these, the left ventricular summit (LVS)—an epicardial region near the bifurcation of the left main coronary artery—is a common site of idiopathic VAs [2]. Despite advances in RFCA techniques, the success rate for LVS‐VAs remains suboptimal, ranging from 20% to 70% [2, 3, 4, 5]. Over recent decades, several strategies have been proposed to improve outcomes in LVS‐VA ablation [6]. Among them, the anatomical approach has been widely adopted. This approach involves delivering RF energy from multiple anatomically adjacent sites—such as the left ventricular (LV) endocardium, coronary cusps, or the right ventricular outflow tract (RVOT)—to reach the arrhythmogenic focus of LVS‐VAs [7, 8]. Nevertheless, achieving adequate lesion depth from the LV endocardium is often crucial for effective ablation in these cases.

Garg et al. recently demonstrated that a prolonged RF ablation strategy starting at a low power of 20 W, with stepwise power titration up to a maximum of 45 W guided by impedance changes and intracardiac echocardiography (ICE), achieved higher success rates than conventional ablation. This strategy, which reached a mean maximum power of 44 W with a median RF application duration of 90 s, did not result in an increase in major complications [9]. This study represents an important prior report demonstrating the efficacy of prolonged RF energy delivery for LVS‐VAs. In addition, experimental studies have shown that prolonged application of RF energy at relatively low power (fixed at ≤ 30 W) can create deeper lesions while minimizing the risk of steam pops [10, 11, 12]. The biophysical rationale for this approach is that prolonged duration at lower power can facilitate conductive heating without excessive tissue overheating. However, clinical data on prolonged‐duration ablation with a fixed low‐power setting remain limited, and its feasibility in real‐world practice has not yet been fully established.

We therefore hypothesized that a fixed low‐power long‐duration RFCA (LPLD RFCA) could safely improve procedural outcomes in LVS‐VAs ablation compared with conventional RFCA. This study aimed to assess the clinical efficacy and safety of the fixed LPLD ablation strategy for the treatment of LVS‐VAs.

## 2 Methods

### 2.1 Study Cohort

Of 440 patients who underwent RFCA for VAs at our institute from January 2013 to June 2025, consecutive patients with LVS‐VAs were included. LVS‐VAs were defined by either of the following criteria:

1. The earliest activation site during the VA was observed in the distal great cardiac vein (GCV), proximal anterior interventricular vein (AIV), or left coronary cusp (LCC), typically with a good pace‐map match.
2. Simultaneous earliest activation was observed on both the epicardium (in the GCV/AIV or LCC) and the LV endocardium, while the pace‐map did not match at any of these sites, indicating intramural LVS sites.

Patients with a reentrant VA were excluded. The indication for RFCA included symptomatic, frequent VAs (VA burden > 10%). Structural heart disease was assessed using 12‐lead electrograms, echocardiography, exercise stress testing, cardiac catheterization, and/or magnetic resonance imaging. We collected data on baseline clinical characteristics, including age, sex, prior ablation history, structural heart disease, left ventricular ejection fraction, laboratory results, and electrocardiographic features of VAs. The myocardial thickness of the LVS was measured using echocardiography. Procedural details, including those of intracardiac electrocardiographic data, outcomes, and complications, were also recorded. The study was approved by the Ethics Committee of Okayama University Graduate School of Medicine, Dentistry, and Pharmaceutical Sciences (K2306‐032). Written informed consent for the ablation procedure and the use of data for research purposes was obtained from all patients. The study was conducted in accordance with the principles of the Declaration of Helsinki.

### 2.2 Electrophysiological Study

Antiarrhythmic drugs were discontinued for a duration exceeding five half‐lives prior to the procedure. All procedures were conducted under conscious sedation with continuous infusion of dexmedetomidine. After achieving successful vascular access, intravenous heparin was administered to maintain a target activated clotting time of > 300 s. Electro‐anatomical mapping was performed using a 3‐dimensional mapping system (CARTO3, Biosense Webster Inc., Irvine, CA, USA). Intracardiac echocardiography (SoundStar, Biosense Webster Inc., Irvine, CA, USA) was utilized to create geometrical imaging of the LV, RV, coronary arteries, and coronary cusps in all patients. A 6Fr decapolar electrode catheter with a lumen (EPstar FIX CS Lumen, Japan Lifeline, Tokyo, Japan) was routinely positioned in the coronary sinus. Additionally, a 2F catheter with a 1.3‐mm electrode length and a 5‐mm interelectrode spacing (EPstar FIX AIV; Japan Lifeline, Tokyo, Japan) was inserted through the lumen and advanced into the GCV/AIV or its branches, such as a communicating vein, as needed. Unipolar and bipolar signals were filtered at ranges of 0.5–200 Hz and 30–500 Hz, respectively. An activation map of the targeted VA was generated using a decapolar or ablation catheter to identify the earliest site of ventricular activation from the LV endocardium, LCC, RVOT, and, if possible, within the GCV/AIV. The local activation time was measured from QRS onset on the 12‐lead ECG to the local bipolar electrogram. Once the earliest VA site was identified, pace mapping was performed with a pacing cycle length equal to that of the spontaneous VA coupling interval. A perfect pace‐map match was defined as a 12/12 lead match, and a good pace‐map match was defined as a 10/12 match. To assess the distance between the ablation site and the coronary arteries, coronary angiography and/or left ventriculography were performed during the procedure in all cases.

### 2.3 RFCA Strategies

A 3.5 mm saline‐irrigated tip catheter (NAVISTAR THERMOCOOL, THERMOCOOL SMARTTOUCH SF, and QDOT MICRO, Biosense Webster Inc., Irvine, CA, USA) was used for ablation. The catheter was inserted into the LV using a retrograde aortic approach. The catheter tip temperature limit was set to 40°C. Generator impedance was measured and recorded for all patients in both groups. The ablation target site on the endocardial surface of the LV was determined based on the prematurity of the local electrogram, specifically selecting the earliest site. If this endocardial earliest site did not correspond to the site closest to the earliest activation site on the epicardial side of the LV, RF applications were delivered on the closest endocardial site as appropriate. Additionally, ablation from the RVOT or LCC was performed as needed, based on proximity to the earliest activation site. In the conventional RFCA strategy, RF energy was delivered at a power setting of 30–40 W. If the VA persisted after multiple applications at the targeted site (typically approximately 10 applications), the conventional RFCA strategy was considered to have failed. In the LPLD‐RFCA strategy, RF energy was delivered at a fixed power setting of 20 W or 25 W for an application duration of 120–180 s. This LPLD setting was consistently applied to all ablation sites on the LV endocardial surface. LPLD RFCA was performed based on the local impedance drop, and if a decrease of 20 Ω or more occurred during ablation, power delivery was discontinued. The decision to apply LPLD RFCA from the outset was based on the prematurity of local potentials and the degree of pace map concordance in the GCV/AIV compared to that on the LV endocardial side during VA.

Acute procedural success was defined as the elimination of clinical VAs, non‐inducibility during an isoproterenol infusion and programmed electrical stimulation, and no recurrence during telemetry ECG monitoring by the time of discharge. Major complications included pericardial effusion, steam pops, heart failure, myocardial infarction, systemic embolism, stroke, major bleeding, and procedure‐related death. Long‐term success was defined as the elimination of clinical VAs or a reduction of more than 90% in the VA burden at the latest follow‐up examination. Patients were followed up through outpatient visits 1–2 months after ablation and subsequently evaluated at 3–6 months intervals. During these visits, the outpatient physician assessed arrhythmia symptoms and performed 12‐lead ECGs, 24 h Holter ECGs, and exercise stress tests. For patients not followed up at our institution, data were collected from referring physicians. Both patients and their referring physicians were contacted directly by telephone or written communication to assess patient outcomes. Follow‐up after the procedure included clinic visits with 12‐lead ECGs and 24‐h Holter ECGs. We compared LVS‐VA outcomes after RFCA, as well as electrocardiographic findings and VA procedural data, between patient groups treated with a fixed LPLD RFCA and conventional RFCA.

### 2.4 Statistical Analysis

Data are expressed as mean ± SD or median and range. Continuous variables were compared using the Student's *t*‐test or Mann–Whitney U test. Categorical variables were analyzed by the *χ* ^2^ or Fisher's exact test, as appropriate. Because Fisher's exact test can be conservative in small or unbalanced samples, Barnard's unconditional exact test was additionally performed to assess the robustness of the findings. Era‐ and catheter‐based subgroup analyses were performed to evaluate the potential impact of historical and technological advancements on procedural outcomes. Univariate logistic regression was performed to identify factors associated with acute procedural success. Sensitivity analyses were performed to address potential selection bias related to intraprocedural crossover. In sensitivity analysis 1, acute success was compared between patients treated with a fixed LPLD RFCA as a primary strategy and those treated with the conventional RFCA only, excluding patients who were switched to the LPLD RFCA during the index procedure after failure of the conventional RFCA. In sensitivity analysis 2, patients who crossed over from the conventional to the LPLD RFCA during the index procedure were analyzed in the conventional group based on the initial ablation strategy. Categorical variables were compared using Fisher's exact test given the small sample size. A *p* < 0.05 was considered statistically significant. All statistical analyses were performed with R software for Windows (version 4.5.0; R Foundation for Statistical Computing; Vienna, Austria).

## 3 Results

### 3.1 Patient Characteristics

**FIGURE 1:**
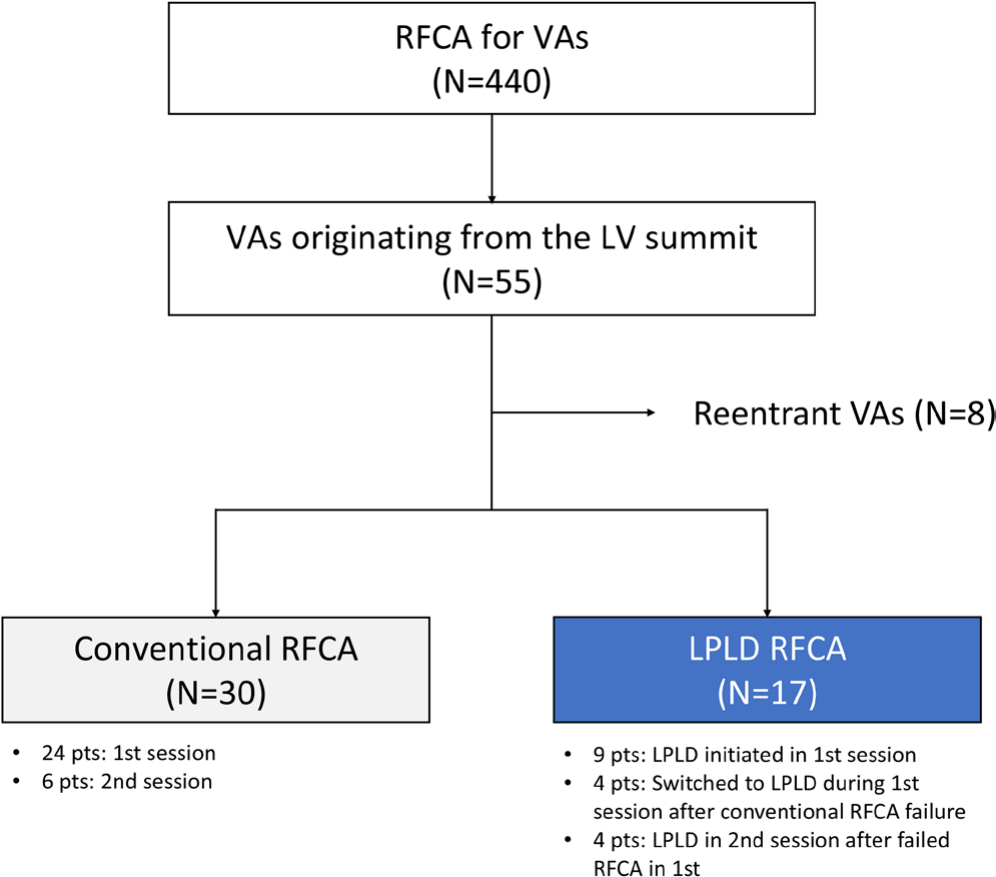
Study Flow Chart.

Of 440 consecutive patients who underwent RFCA for VAs, 47 with LVS‐VAs were included in this study. Of these, 30 patients underwent conventional RFCA, while 17 underwent LPLD RFCA (Figure 1). Of the 17 patients in the LPLD RFCA group, 13 were treated with this strategy from the start of the RF application, whereas the remaining 4 were switched to LPLD RFCA because of persistent VA refractory to the conventional RFCA strategy. Four of the 13 patients that underwent LPLD RFCA had previously undergone conventional RFCA in their first session but experienced treatment failure or VA recurrence. The clinical characteristics of the study population are summarized in Table 1. The mean age of the patients was 60 ± 19 years, and 35 patients (75%) were male. The mean LV ejection fraction was 52% ± 14%, and myocardial thickness at LVS was 12 ± 2.4 mm. The mean VA burden was 28% ± 10% of total heartbeats. Structural heart disease was present in 19 patients (40%), including dilated cardiomyopathy in 10 patients (21%), ischemic heart disease in 5 (11%), hypertrophic cardiomyopathy in 2 (4%), post‐myocarditis in 1 (2%), and a ventricular septal defect in 1 (2%). The remaining 28 patients (60%) had no significant structural heart disease. Ten patients (21%) had previously undergone an unsuccessful RFCA procedure for the same VA. Prior to the procedure, beta‐blockers and/or other antiarrhythmic drugs were administered to 38 patients (81%) and 7 patients (15%), respectively. There were no significant differences in the baseline clinical characteristics between the two groups.

**TABLE 1:** Patient characteristics.

	Overall (*n* = 47)	LPLD RFCA (*n* = 17)	Conventional RFCA (*n* = 30)	*p*
Age, y	60 ± 19	57 ± 22	63 ± 17	0.19
Male	35 (75%)	13 (77%)	22 (73%)	1.00
BMI, kg/m^2^	26 ± 5.3	25 ± 3.7	27 ± 6.0	0.39
Structural heart disease	19 (40%)	7 (41%)	12 (40%)	1.00
Dilated cardiomyopathy	10 (21%)	3 (18%)	7 (23%)	—
Ischemic heart disease	5 (11%)	3 (18%)	2 (7%)	—
Hypertrophic cardiomyopathy	2 (4%)	0 (0%)	2 (7%)	—
Post‐Myocarditis	1 (2%)	0 (0%)	1 (3%)	—
Ventricular septal defect	1 (2%)	1 (6%)	0 (0%)	—
LVEF, %	52 ± 14	51 ± 14	54 ± 13	0.92
Myocardial thickness at LV summit, mm	12 ± 2.4	12 ± 1.8	12 ± 2.7	0.97
VA burden before ablation, %	28 ± 10	27 ± 9	28 ± 11	0.93
Multiple VAs	17 (36%)	8 (47%)	9 (30%)	0.39
Sustained ventricular tachycardia	5 (11%)	1 (6%)	4 (13%)	0.76
Previous VA ablation	10 (21%)	4 (24%)	6 (20%)	1.00
Pre‐procedural CMR scan	42 (89%)	15 (88%)	27 (90%)	1.00
Late gadolinium enhancement	10 (23%)	4 (25%)	6 (22%)	0.40
Antiarrhythmic drug usage	39 (83%)	13 (77%)	26 (87%)	0.62
β‐blocker	38 (81%)	12 (71%)	26 (87%)	0.34
Class I, III, or IV	7 (15%)	3 (18%)	4 (13%)	1.00

*Note:* Values are presented as *n* (%), or mean ± standard deviation (SD).

Abbreviations: AAD, antiarrhythmic drug; BMI, body mass index; BNP, brain natriuretic peptide; CMR, cardiac magnetic resonance; LGE, late gadolinium enhancement; LV, left ventricle; LVEF, left ventricular ejection fraction; NICM, non‐ischemic cardiomyopathy; RFCA, radiofrequency catheter ablation; VA, ventricular arrhythmia; VT, ventricular tachycardia.

### 3.2 Electrocardiographic Characteristics of VAs

The electrocardiographic characteristics of the VAs are summarized in Table 2. On the 12‐lead ECG, the morphology of the clinical VA showed a right bundle branch block pattern in 31 patients (66%) and a left bundle branch block pattern in 16 (34%). A left inferior axis was observed in 10 patients (21%), whereas a right inferior axis was found in 37 (79%). The mean QRS duration was 155 ± 19 ms. The precordial transition occurred in lead V2 in 5 patients (11%) and in lead V3 in 10 (22%). A positive concordant pattern in the precordial leads was observed in 7 patients (15%). The maximum deflection index of the VAs was significantly larger in the LPLD RFCA group compared with the conventional RFCA group (0.59 ± 0.12 vs. 0.50 ± 0.12, *p* = 0.02). No significant differences were observed between the two groups for other ECG parameters.

**TABLE 2:** Electrocardiographic characteristics of ventricular arrhythmia.

	Overall (*n* = 47)	LPLD RFCA (*n* = 17)	Conventional RFCA (*n* = 30)	*p*
QRS duration, ms	155 ± 19	154 ± 16	155 ± 20	0.92
RBBB/LBBB pattern	31/16 (66%/34%)	11/6 (65%/35%)	20/10 (67%/33%)	1.00
Right inferior/Left inferior axis	37/10 (79%/21%)	15/2 (88%/12%)	22/8 (73%/27%)	0.41
Precordial transition				
Positive concordance	7 (15%)	2 (12%)	5 (17%)	0.98
V2	5 (11%)	2 (12%)	3 (10%)	0.92
V3	10 (22%)	3 (18%)	7 (24%)	—
Maximum deflection index	0.54 ± 0.1	0.59 ± 0.12	0.50 ± 0.12	0.02
R‐wave ratio in lead III/II	1.1 ± 0.1	1.1 ± 0.1	1.1 ± 0.2	0.73
Q‐wave ratio in lead aVL/aVR	1.3 ± 0.5	1.4 ± 0.4	1.3 ± 0.5	0.57
R‐wave amplitude in II, mV	2.1 ± 0.6	2.2 ± 0.6	2.0 ± 0.5	0.33
R‐wave amplitude in III, mV	2.2 ± 0.7	2.4 ± 0.8	2.2 ± 0.6	0.37
Q‐wave amplitude in aVR, mV	1.0 ± 0.3	1.0 ± 0.3	1.0 ± 0.3	0.73
Q‐wave amplitude in aVL, mV	1.3 ± 0.4	1.3 ± 0.4	1.2 ± 0.4	0.39
QS pattern in lead I	12 (26%)	5 (29%)	7 (23%)	0.92
S‐wave in lead V6	13 (28%)	6 (35%)	7 (23%)	0.59

*Note:* Values are presented as *n* (%) or mean ± standard deviation (SD).

Abbreviations: LBBB, left bundle branch block; RBBB, right bundle branch block.

### 3.3 Mapping and Ablation Procedures

The earliest activation site across the entire heart was located in the GCV/AIV in 37 patients (79%), the LCC in 6 (13%), and simultaneous earliest activation at multiple sites in 4 (9%). In all four patients whose earliest activation site was in the LV endocardium, the local electrogram at that site was either isochronal with or occurred after the QRS onset. A QS pattern on the unipolar electrogram at the earliest activation site in the LV endocardium was observed in 11 patients (65%) treated with LPLD RFCA and 18 (62%) treated with conventional RFCA, with no significant differences between the groups. A good pace‐map match at the ablation site was observed in one patient (7%) in the LPLD RFCA group and in 7 patients (24%) in the conventional RFCA group. At the earliest activation site, the local electrogram preceded the QRS onset by 21 ± 10 ms in the LPLD RFCA group and by 24 ± 10 ms in the conventional RFCA group (*p* = 0.32), indicating no significant difference between the two groups. At the LV endocardial site selected as the ablation target, the local electrogram preceded the QRS onset in both groups but occurred significantly later in the LPLD RFCA group (8 ± 8 ms vs. 16 ± 13 ms, *p* = 0.02) (Table 3). Table 4 summarizes the procedural details. In the LPLD RFCA group, RF applications were delivered from the LCC in 10 patients (59%) and from the RVOT in 4 (24%), in addition to ablation from the LV endocardium. In the conventional RFCA group, RF applications were delivered from the LCC in 13 patients (43%), the RVOT in 3 (10%), and the GCV/AIV in 5 (17%), in addition to ablation from the LV endocardium. Regarding the ablation catheters used, the distribution of catheter type was similar between the two groups (*p* = 0.38). The THERMOCOOL STSF catheter was used in the vast majority of cases in both groups (94% in LPLD vs. 87% in conventional, *p* = 0.64). The mean RF power was significantly lower in the LPLD RFCA group than in the conventional RFCA group (20 ± 1.0 W vs. 36 ± 5.4 W, *p* < 0.01). There were no significant differences in the number of RF applications (15.4 ± 8.0 vs. 16.1 ± 11.1, *p* = 0.82) or total energy delivery (*p* = 0.58) between the two groups. Compared with the conventional group, the LPLD group demonstrated significantly higher energy delivery per individual application (2391 ± 798 J vs. 1651 ± 588 J, *p* = 0.01) and a significantly longer total RF application time (30 ± 13 min vs. 15 ± 11 min, *p* < 0.01). Baseline generator impedance (126 ± 11 Ω vs. 133 ± 15 Ω, *p* = 0.09) and maximum impedance drop (14.9 ± 3.2 Ω vs. 14.6 ± 3.1 Ω, *p* = 0.77) were comparable between the two groups. Furthermore, RF application time per session was significantly longer in the LPLD group (169 ± 16 s vs. 55 ± 15 s, *p* < 0.01). The overall procedure duration was also longer in the LPLD RFCA group (259 ± 63 min vs. 221 ± 43 min, *p* = 0.03).

**TABLE 3:** Electrophysiological characteristics of ventricular arrhythmias.

	Overall (*n* = 47)	LPLD RFCA (*n* = 17)	Conventional RFCA (*n* = 30)	*p*
Earliest activation site				0.13
GCV/AIV	37 (79%)	15 (88%)	22 (73%)	—
LCC	6 (13%)	0 (0%)	6 (20%)	—
Simultaneous earliest activation at multiple sites	4 (9%)	2 (12%)	2 (7%)	—
Earliest activation time preceding QRS complex, ms	23 ± 10	21 ± 10	24 ± 10	0.32
Local activation time preceding QRS complex at the ablation site, ms	13 ± 12	8 ± 8	16 ± 13	0.02
Unipolar QS pattern at the ablation site	29 (63%)	11 (65%)	18 (62%)	1.00
Pace‐map match at the ablation site on the LV endocardium				
Good/Perfect	8/0 (17%/0%)	1/0 (6%/0%)	7/0 (24%/0%)	0.26

*Note:* Values are presented as *n* (%) or mean ± standard deviation (SD).

Abbreviations: AIV, anterior interventricular vein; GCV, great cardiac vein; LCC, left coronary cusp; LV, left ventricle; VA, ventricular arrhythmia.

**TABLE 4:** Ablation details and outcomes.

	Overall (*n* = 47)	LPLD RFCA (*n* = 17)	Conventional RFCA (*n* = 30)	*p*
Ablation sites				
LV endocardium	47 (100%)	17 (100%)	30 (100%)	NS
LCC	23 (49%)	10 (59%)	13 (43%)	0.47
RVOT	7 (15%)	4 (24%)	3 (10%)	0.49
GCV	5 (12%)	0 (0%)	5 (17%)	0.20
Ablation catheter				0.38
NAVISTAR THERMOCOOL	3 (7%)	0 (0%)	3 (10%)	—
THERMOCOOL STSF	42 (89%)	16 (94%)	26 (87%)	0.64
QDOT MICRO	2 (4%)	1 (6%)	1 (3%)	—
Intracardiac echocardiography usage, *n* (%)	47 (100%)	15 (100%)	27 (100%)	NS
RF power, W	30 ± 8.7	20 ± 1.0	36 ± 5.4	< 0.01
Number of RF application	15.8 ± 10.1	15.4 ± 8.0	16.1 ± 11.1	0.82
Total ablation energy, Joules	29510 ± 19000	27278 ± 21672	31612 ± 13651	0.58
Ablation energy per application, Joules	1925 ± 757	2391 ± 798	1651 ± 588	0.01
Baseline generator impedance, Ω	130 ± 14	126 ± 11	133 ± 15	0.09
Impedance drop, Ω	14.1 ± 3.1	14.9 ± 3.2	14.6 ± 3.1	0.77
Total RF application time, min	21 ± 14	30 ± 13	15 ± 11	< 0.01
RF application time per session, sec	97 ± 58	169 ± 16	55 ± 15	< 0.01
Procedure time, min	236 ± 54	259 ± 63	221 ± 43	0.03
Acute procedural success	32 (68%)	15 (88%)	17 (56%)	0.048^a^
Procedural complications	0 (0%)	0 (0%)	0 (0%)	1
Long‐term success	27 (57%)	13 (77%)	14 (47%)	0.067^a^

*Note:* Values are presented as *n* (%) or mean ± standard deviation (SD).

Abbreviations: GCV, great cardiac vein; LCC, left coronary cusp; LV, left ventricle; RF, radiofrequency; RVOT, right ventricular outflow tract.

^a^ Fisher's exact test.

### 3.4 Outcomes and Complications

**FIGURE 2:**
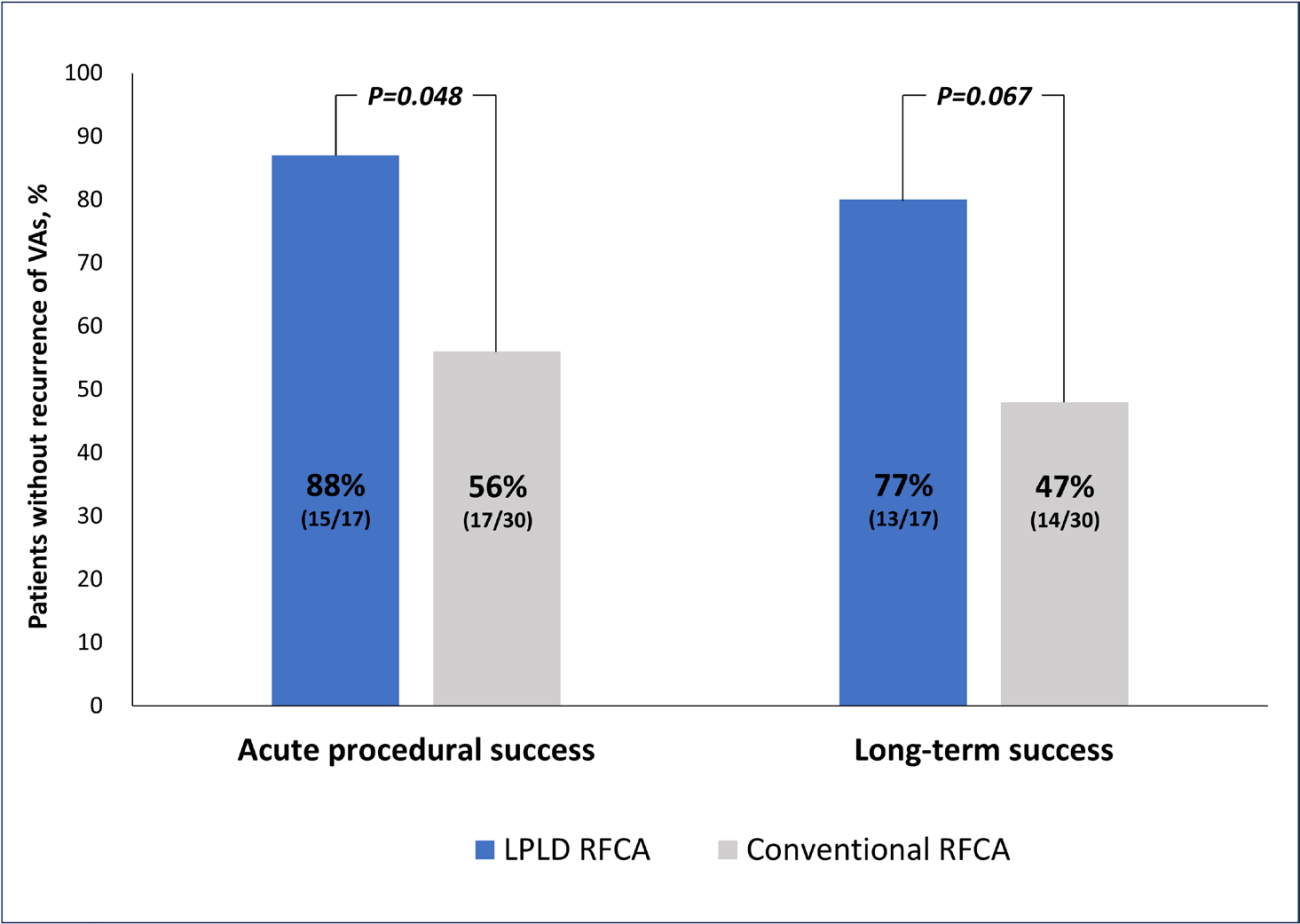
Acute and Long‐Term RFCA Outcomes.

**FIGURE 3:**
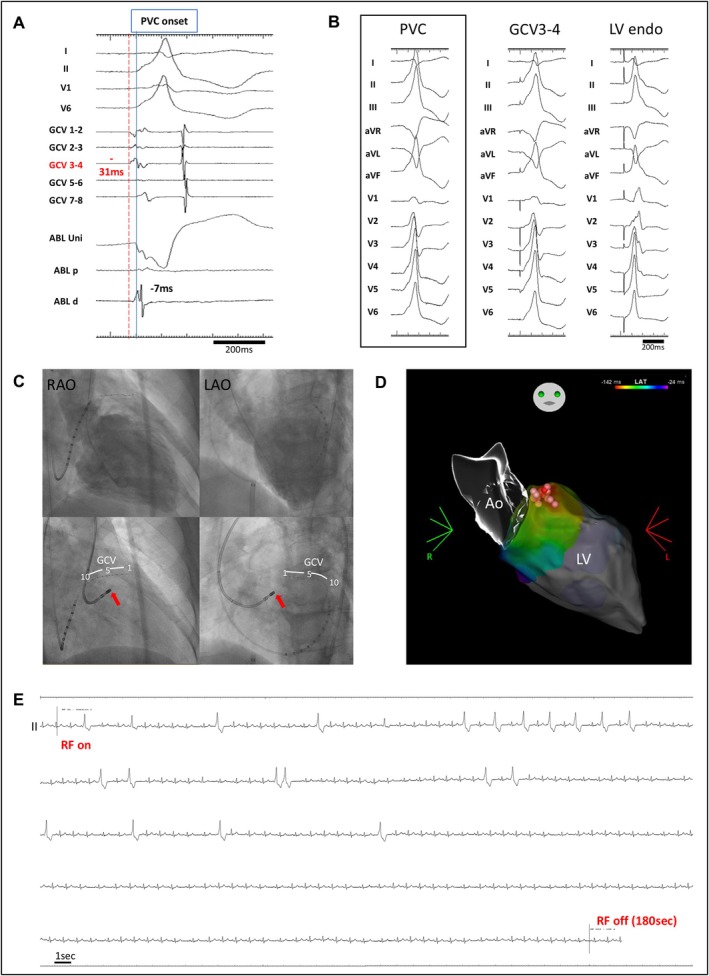
Representative case (2) with low‐power long‐duration (LPLD) ablation for LVS‐VAs (A) Intracardiac recordings and (B) pace mapping in the great cardiac vein (GCV) and the successful ablation site on the left ventricular (LV) endocardial surface. The earliest activation was recorded at the distal GCV (GCV 3–4), occurring 31 ms before the onset of the QRS complex on the surface electrocardiogram. An excellent pace map was obtained at this site. In contrast, at the successful ablation site on the LV endocardial surface, activation was delayed by 24 ms compared to GCV 3–4, and the pace map did not match the morphology of the target premature ventricular contraction (PVC) morphology. (C) Fluoroscopic images of the left ventriculography (upper panels) and catheter positioning (lower panels) in the right anterior oblique (RAO) and left anterior oblique (LAO) views. The ablation catheter at the successful LV endocardial site is indicated by a red arrow. (D) A three‐dimensional electroanatomic mapping image showing the activation map during the target PVC and the ablation sites (circular tags in a reddish color scheme) on the LV endocardial surface (E) Continuous ECG recording during the first radiofrequency (RF) application at 20 W from the LV endocardial surface. The target PVC disappeared approximately 100 s after RF application initiation. The RF delivery was maintained at 20 W for up to 180 s, followed by additional ablation in the surrounding area of the LV endocardial surface. The total RF application time was 38 min. Thereafter, the target PVC could not be induced by programmed stimulation and drug administration.

**FIGURE 4:**
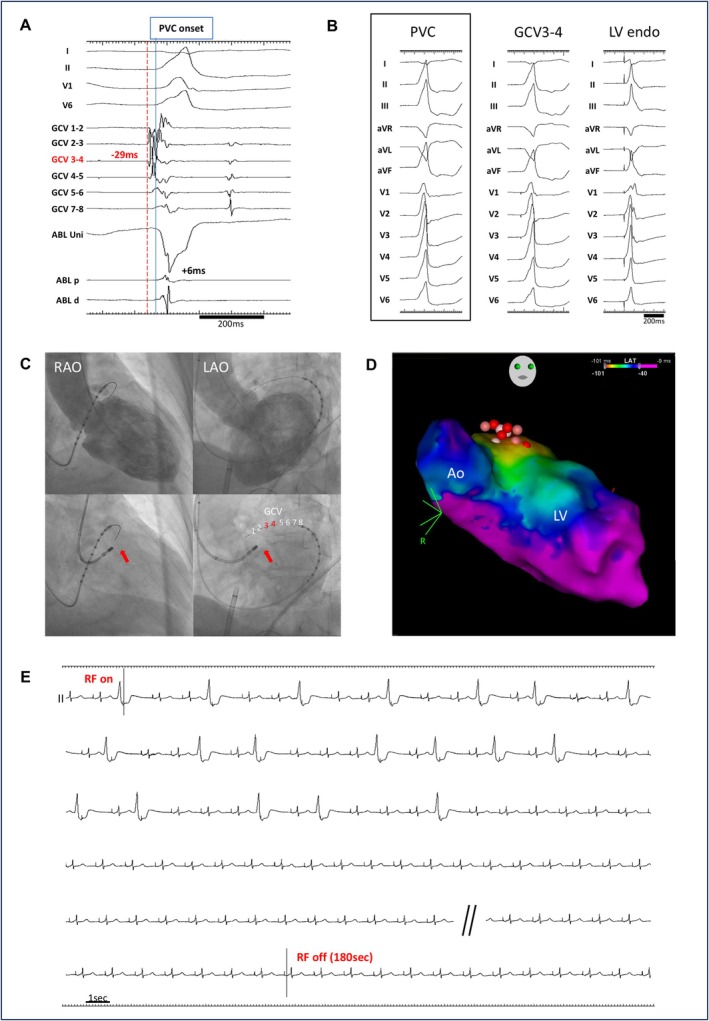
Representative case (13) with low‐power long‐duration (LPLD) ablation for an LVS‐VA (A) Intracardiac recordings and (B) pace mapping in the great cardiac vein (GCV) and the successful ablation site on the left ventricular (LV) endocardial surface. The earliest activation was recorded at the distal GCV (GCV 3–4), occurring 29 ms before the onset of the QRS complex on the surface electrocardiogram. An excellent pace map was obtained with a stimulus‐to‐QRS delay at this site. At the successful ablation site on the LV endocardial surface, local activation was delayed by 6 ms from the onset of the QRS complex, and the pace map did not match the morphology of the target premature ventricular contraction (PVC) morphology. (C) Fluoroscopic images of left ventriculography (upper panels) and catheter positioning (lower panels) in right anterior oblique (RAO) and left anterior oblique (LAO) views. The ablation catheter at the successful LV endocardial site is indicated by a red arrow. (D) A three‐dimensional electroanatomic mapping image showing the activation map during the target PVC and the ablation sites (circular tags in a reddish color scheme) on the LV endocardial surface. (E) Continuous ECG recording during the first radiofrequency (RF) application at 20 W from the LV endocardial surface. The target PVC disappeared approximately 45 s after RF application initiation. RF delivery was maintained at 20 W for up to 180 s, followed by additional ablation at up to 25 W in the surrounding areas, including the LV endocardial surface and left coronary cusp. The total RF application time was 38 min. Thereafter, the target PVC could no longer be induced by programmed stimulation and drug administration.

Table 4 also shows the outcomes of RFCA. Acute procedural success was achieved in 15 of the 17 patients (88%) in the LPLD RFCA group and in 17 of 30 patients (56%) in the conventional RFCA group (*p* = 0.048 and 0.028 by Fisher's exact test and Barnard's exact test, respectively). At a median follow‐up of 16 months (range: 8–30 months), VA recurrence was observed in 2 patients in the LPLD RFCA group and 3 patients in the conventional RFCA group; no patients in either group achieved late success. Consequently, long‐term success was observed in 13 of 17 patients (77%) in the LPLD RFCA group and in 14 of 30 patients (47%) in the conventional RFCA group (*p* = 0.067 and 0.055 by Fisher's exact test and Barnard's exact test, respectively) (Figure 2). The distribution of the two ablation strategies differed significantly across the three eras (*p* < 0.001), with the LPLD strategy being performed exclusively in the contemporary era (2018–2025), whereas the conventional RFCA was used throughout the study period (Table S1 and Figure S1). Analysis by era revealed that the acute success rate in the conventional group improved from 43% in 2013–2017 to 69% in 2018–2021 and 67% in 2022–2025. While this trend suggested technological progress, the difference between the early (2013–2017) and late (2018–2025) conventional groups was not statistically significant (*p* = 0.27). Notably, in a comparison within the contemporary era (2018–2025), the LPLD group (all performed between 2020 and 2025) maintained numerically higher success rates compared to the conventional group for both acute procedural success (88% vs. 69%, *p* = 0.22) and long‐term success (76% vs. 56%, *p* = 0.28). Similarly, in the subgroup analysis of patients treated with the THERMOCOOL STSF catheter, the LPLD group demonstrated higher success rates than the conventional group for both acute (88% vs. 58%, *p* = 0.08) and long‐term outcomes (75% vs. 50%, *p* = 0.19) (Table S1 and Figure S1). No major procedural complications occurred during RFCA, and a local impedance drop of ≥ 20 Ω was not observed in any case during RF application. The electrophysiological measurements, ablation details, and outcomes for each patient in the LPLD RFCA group are summarized in Table 5. Representative cases demonstrating acute success in the LPLD RFCA group are presented in Figures 3 and 4. The results of univariate logistic regression analysis for predictors of acute procedural success are presented in Table 6; LPLD RFCA was the only variable significantly associated with acute procedural success in patients with LVS‐VAs. In sensitivity analysis 1, which excluded patients who were switched to LPLD RFCA during the index procedure after failure of conventional RFCA, the primary LPLD group continued to demonstrate a higher acute success rate than the conventional‐only group (92% vs. 57%, *p* = 0.033). Similarly, in sensitivity analysis 2, patients who crossed over from conventional to LPLD RFCA during the index procedure were analyzed in the conventional group based on the initial ablation strategy. In this analysis, the acute success rate also remained higher in the LPLD group (92% vs. 50%, *p* = 0.008), consistent with the primary analysis (Table S1).

**TABLE 5:** Details of the electrophysiological measurements, ablation procedures, and outcomes for each patient in the LPLD RFCA Group.

Case No.	Age (y)	Sex	SHD	Activation time at the GCV/AIV (ms)	Activation time at the ablation site (ms)	Pace‐map match at the ablation site in the LV	Mean RF power (W)	Ablation energy per session (J)	Max RF power (W)	RF application time (min)	Average application time per ablation (sec)	Maximum LI drop during ablation (ohm)	Ablation sites	Acute success	Long‐term success
1	45	M	None	−10	−3	Good	20	1374	30	20	63	11	LV endo, LCC, RVOT	+	+
2	72	M	DCM	−31	−7	Not match	20	2917	25	28	153	18	LV endo, LCC	+	+
3	80	F	None	−15	7	Not match	20	3203	20	17	170	14	LV endo	+	+
4	69	M	DCM	−26	−3	Not match	20	3324	20	32	175	18	LV endo	+	+
5	79	M	None	−30	−21	Not match	20	2735	25	21	140	18	LV endo, LCC	+	+
6	21	M	None	−13	−10	Not match	20	2244	20	40	120	17	LV endo	+	+
7	60	F	None	−21	−15	Not match	20	2192	30	41	112	12	LV endo, LCC, RVOT	+	—
8	58	M	IHD	−21	−13	Not match	20	1960	30	49	98	18	LV endo, LCC	—	—
9	27	M	None	−23	−7	Not match	20	3525	20	18	180	19	LV endo	+	+
10	68	M	None	−21	−10	Not match	20	2698	20	37	148	18	LV endo	—	—
11	75	M	IHD	−26	−7	Not match	21	2944	25	33	152	13	LV endo, LCC	+	+
12	22	F	VSD	−23	−10	Not match	20	1679	30	17	85	12	LV endo, LCC	+	+
13	79	M	None	−29	6	Not match	20	2710	30	38	134	13	LV endo, LCC	+	+
14	60	M	DCM	−26	−15	Not match	20	3180	20	30	164	17	LV endo	+	+
15	37	F	None	−50	−13	Not match	20	1747	30	21	90	10	LV endo, RVOT	+	+
16	18	M	None	−10	−10	Not match	20	1567	25	12	78	11	LV endo, LCC	+	+
17	69	M	ICM	−26	−18	Not match	24	2521	35	62	100	13	LV endo, LCC, RVOT	+	+

*Note:* Values are presented as *n* (%) or mean ± standard deviation (SD).

Abbreviations: AIV, anterior interventricular vein; DCM, dilated cardiomyopathy; GCV, great cardiac vein; IHD, ischemic heart disease; LCC, left coronary cusp; LI, local impedance; LV, left ventricle; RF, radiofrequency; RVOT, right ventricular outflow tract; SHD, structural heart disease; VSD, ventricular septal defect.

**TABLE 6:** Univariate logistic regression analysis of acute procedural success.

Variable	Univariate analysis		
	Odds ratio	95% CI	*p*
Age	1.01	0.98–1.05	0.30
Male	1.79	0.44–7.01	0.40
LVEF	1.00	0.96–1.05	0.91
Antiarrhythmic drug usage	0.67	0.09–3.38	0.65
Structural heart disease	1.03	0.30–3.72	0.97
LBBB morphology	0.37	0.07–1.43	0.17
Earliest activation time preceding QRS complex	0.99	0.93–1.05	0.64
Local activation time preceding QRS complex at the ablation site	1.01	0.96–1.07	0.62
Maximum deflection index	2.44	0.02–328	0.71
Myocardial thickness at LV summit	0.87	0.63–1.14	0.36
Total RF energy	1.00	0.99–1.00	0.58
LPLD RFCA	5.74	1.30–40.6	0.04

Abbreviations: CI, confidence interval; LBBB, left bundle branch block; LPLD, low‐power long‐duration; LVEF, left ventricular ejection fraction; RFCA, radiofrequency catheter ablation.

## 4 Discussion

This study evaluated the efficacy and safety of the endocardial fixed low‐power long‐duration RFCA (LPLD RFCA), defined as an RF delivery at 20 W or 25 W for 120–180 s, for the treatment of LVS‐VAs. Compared with conventional RFCA, the LPLD RFCA showed a significantly higher acute success rate (88% vs. 56%) without major procedural complications. By avoiding the procedural risks associated with epicardial, intra‐coronary venous system, or high‐power ablation, the fixed LPLD strategy may represent a safer and more effective therapeutic alternative.

LVS‐VAs account for approximately 40% of LV outflow tract arrhythmias and remain challenging to treat because of structural barriers, myocardial wall thickness, and limited catheter accessibility [2, 3, 13, 14, 15]. Although epicardial ablation can be effective, it is often limited by proximity to coronary arteries, epicardial fat, and procedural risks [4, 16]. Alternative strategies—such as bipolar or simultaneous unipolar ablation, ethanol injection into adjacent coronary veins, and the use of half‐saline irrigation—have been proposed [17, 18, 19, 20], but their efficacy depends highly on operator expertise and institutional resources. Consequently, anatomical ablation from adjacent endocardial sites, including the coronary cusps, RVOT, mitral annulus, and LV endocardium, has become a widely adopted approach [7, 8, 21, 22, 23]. However, a recent report has demonstrated that efficacy of anatomic ablation declines when the anatomical distance between the target and earliest activation sites exceeds approximately 13 mm [24]. Deeper lesion formation from the LV endocardium can thus be critical for successful treatment even with anatomical ablation. Our findings suggest that LPLD RFCA, which delivers prolonged RF energy at a fixed low‐power setting from the LV endocardium, may facilitate effective lesion depth even in arrhythmias with presumed epicardial or intramural origins and achieve procedural success rates comparable to those reported in multicenter studies that included epicardial ablation [23]. Notably, nearly half of the patients in the LPLD group had previously failed conventional RFCA. Moreover, the LPLD group exhibited a significantly larger maximum deflection index and earlier local activation time in the GCV/AIV, suggesting that those VAs had more epicardial origins. These observations support the potential utility of LPLD RFCA in anatomically challenging LVS‐VAs, although direct assessment of lesion depth was not available. Importantly, the robustness of these findings was supported by sensitivity analyses addressing potential selection bias related to intraprocedural crossover. The higher acute success rate associated with the LPLD strategy persisted after excluding patients who were switched to LPLD RFCA during the index procedure following failure of conventional RFCA. In addition, when crossover patients were analyzed in the conventional group based on the initial ablation strategy, the direction and magnitude of the association remained consistent with the primary analysis. These results suggest that the observed benefit of LPLD RFCA is not solely driven by rescue or crossover cases, but rather reflects the effectiveness of applying the LPLD strategy itself.

The biophysics of RF lesion formation involves two primary mechanisms: resistive heating at the electrode–tissue interface and conductive heating that spreads deeper into the myocardium. Earlier studies using non‐irrigated catheters showed that lesion growth plateaus after approximately 20 s [25], whereas studies using irrigated‐tip catheters demonstrated continued lesion expansion with prolonged RF applications due to enhanced conductive heating. Rogers et al. reported that lesion volume and depth increased linearly with ablation duration beyond the first minute in bovine myocardium [12]. While some in vitro models have suggested that the rate of lesion expansion significantly slows down after approximately 90 s [10], the LVS region is characterized by significant myocardial thickness and the presence of adjacent large vessels that act as a “heat sink.” These anatomical factors may necessitate more prolonged energy delivery to achieve adequate depth. Furthermore, as Borne et al. indicated that lesion size is more proportional to power than to duration [11], the choice of energy delivery parameters is critical.

High‐power (> 40 W), long‐duration ablation can therefore create large and deep lesions but carries an increased risk of complications such as steam pops. In our study, although the total energy delivery was comparable between the two groups, the energy delivered per individual application was significantly higher in the LPLD group. This sustained energy delivery at a fixed low‐power setting was intended to maximize the conductive heating phase while remaining below the thermal threshold for localized tissue overheating. By contrast, prolonged ablation at low‐power settings may allow for more gradual lesion formation while mitigating excessive thermal injury. Garg et al. recently demonstrated improved procedural success using prolonged‐duration ablation for LVS‐VAs with a power titration strategy reaching a mean maximum power of 44 W, without a significant difference in major complication rates compared with conventional ablation [9]. Our study focused on a fixed lower power strategy (20‐25 W) with even further prolonged duration, resulting in favorable acute outcomes without major complications. While direct comparisons between these approaches are not possible, our findings suggest that a standardized fixed LPLD protocol may represent a feasible and reproducible alternative strategy for managing these challenging substrates. The distribution of ablation strategies significantly shifted toward the LPLD strategy in the contemporary era (*p* < 0.001; Table S1 and Figure S1). To address potential confounding factors associated with the long study period, we analyzed whether the superior success rate of LPLD RFCA was influenced by historical or technological advancements. Our era‐based analysis showed that while the success rate in the conventional group improved in the more recent era, the LPLD group consistently demonstrated a numerically higher success rate even when compared with the contemporary conventional group. Furthermore, the distribution of catheter types was similar between the two groups, and the superior outcome of LPLD RFCA was observed even when restricted to patients treated with the same contemporary catheter (THERMOCOOL STSF). Notably, within the contemporary era (2018–2025) and among patients treated with the same contemporary catheter technology, the LPLD group consistently demonstrated numerically higher success rates for both acute and long‐term outcomes. In addition, comparable generator impedance drops suggested that the quality of catheter–tissue contact was similar between the groups. However, incorporating real‐time contact force data and lesion indices, such as the Ablation Index, in future research will likely provide more granular insights into the biophysical advantages of the LPLD strategy. These consistent findings across different procedural eras and catheter technologies suggest that the observed clinical benefit is primarily derived from the LPLD strategy itself. Although it is uncertain whether all patients who benefited from LPLD RFCA would have failed conventional RFCA, our findings suggest that this approach achieves a favorable balance between efficacy and safety. On the other hand, LPLD RFCA requires longer RF application and overall procedure times, which may be a practical limitation. Further prospective studies with larger populations and detailed lesion imaging are needed to confirm the optimal energy–time parameters and assess the generalizability of this approach.

## 5 Limitations

This study has some limitations. First, it was a single‐center, observational study with a small sample size, which may limit the generalizability of the findings. The limited number of patients, particularly in the subgroup and sensitivity analyses, may have also restricted the statistical power to detect significant differences in those specific comparisons. Second, variations in VA origin and cardiac geometry, as well as inter‐operator differences, may have influenced the results. Third, the retrospective and non‐randomized design and the significant historical shift in ablation strategy over the 12‐year study period (*p* < 0.001) introduce potential selection and historical biases. The choice of strategy—whether LPLD was used as a primary approach or a rescue therapy—was partially influenced by operator judgment and coincided with general improvements in mapping and catheter technologies. While we addressed this by performing era‐matched and catheter‐matched subgroup analyses—which yielded consistent numerical trends across both acute and long‐term outcomes—the inherent confounding associated with technological evolution cannot be entirely eliminated. Although our sensitivity analyses showed consistent results, residual confounding related to the selection process may remain. Prospective, randomized studies with larger sample sizes and more detailed analyses are necessary to confirm our results. Lastly, although the clinical efficacy of the LPLD strategy was favorable, no imaging or histological data were available to directly assess lesion depth or transmurality. Therefore, the proposed mechanism that prolonged‐duration ablation at low power results in deeper lesion formation remains speculative and is inferred only from clinical outcomes. In this context, we were also unable to directly determine whether LPLD RFCA creates deeper lesions compared with conventional RFCA. Although direct verification of lesion depth in the clinical setting is inherently challenging, further investigations into the biophysics of radiofrequency ablation are warranted to elucidate the underlying mechanisms.

## 6 Conclusions

Compared with conventional RFCA, endocardial RFCA using a fixed LPLD strategy demonstrated a significantly higher rate of acute procedural success in the treatment of LVS‐VAs. This approach may represent a feasible alternative ablation strategy for LVS‐VAs. Further large‐scale, randomized studies are warranted to confirm these findings and clarify the underlying biophysical mechanisms.

## Funding

The authors have nothing to report.

## Ethics Statement

The present study was approved by the Ethics Committee of Okayama University Graduate School of Medicine, Dentistry, and Pharmaceutical Sciences (K2306‐032).

## Consent

Patients had consented to the use of their data for research purposes through an opt‐out method.

## Conflicts of Interest

H.M. and N.N. are affiliated with the department endowed by Medtronic Japan Co. Ltd. The other authors declare no conflicts of interest.

## Supporting information


**Figure S1:** Temporal distribution of ablation strategies and subgroup analyses of procedural success. (A) Distribution of ablation cases by era (2014–2017, 2018–2021, and 2022–2025). The clinical application of the LPLD strategy significantly increased in the contemporary era (p < 0.001). (B) Comparison of acute and long‐term procedural success rates between the LPLD and conventional groups in the contemporary era (2018–2025). (C) Comparison of success rates in the subgroup treated exclusively with the THERMOCOOL STSF catheter. Although the comparisons in (B) and (C) did not reach statistical significance due to the limited sample size, the LPLD group demonstrated numerically higher success rates for both acute and long‐term outcomes.
**Table S1:** Comparison of procedural outcomes and success rates stratified by treatment era and ablation catheter type.

## Data Availability

Data available on request due to privacy/ethical restrictions.
