# Supplementary material for: Efficacy of Fixed Low‐Power Long‐Duration Radiofrequency Ablation of Ventricular Arrhythmias Originating From the Left Ventricular Summit
Source: J Arrhythm. 2026 May 3;42(3):e70339. doi: 10.1002/joa3.70339 (PMC13136689; doi:10.1002/joa3.70339)
Supplement: Supplementary file 1 — Figure S1: Temporal distribution of ablation strategies and subgroup analyses of procedural success. (A) Distribution of ablation cases by era (2014–2017, 2018–2021, and 2022–2025). The clinical application of the LPLD strategy significantly increased in the contemporary era (p < 0.001). (B) Comparison of acute and long‐term procedural success rates between the LPLD and conventional groups in the contemporary era (2018–2025). (C) Comparison of success rates in the subgroup treated exclusively with the THERMOCOOL STSF catheter. Although the comparisons in (B) and (C) did not reach statistical significance due to the limited sample size, the LPLD group demonstrated numerically higher success rates for both acute and long‐term outcomes. Table S1: Comparison of procedural outcomes and success rates stratified by treatment era and ablation catheter type. [file JOA3-42-e70339-s001.zip › joa370339-sup-0002-Revised Supplemental Table 1.docx]

**Supplemental Table 1**

| Supplemental Table 1. Comparison of procedural outcomes and success rates stratified by treatment era and ablation catheter type. | | | | |
| --- | --- | --- | --- | --- |
|  | Overall | LPLD RFCA | Conventional RFCA | P-value |
| Era of catheter ablation |  |  |  | <0.001 |
| 2014 - 2017 | 14 (30%) | 0 (0%) | 14 (47%) |  |
| 2018 - 2021 | 20 (43%) | 7 (41%) | 13 (43%) |  |
| 2022 - 2025 | 13 (27%) | 10 (59%) | 3 (10%) |  |
| Acute procedural success |  |  |  |  |
| Contemporary era (2018-2025) | 26/33 (79%) | 15/17 (88%) | 11/16 (69%) | 0.22 |
| SMARTTOUCH STSF catheter | 29/42 (69%) | 14/16 (88%) | 15/26 (58%) | 0.08 |
| Long-term success |  |  |  |  |
| Contemporary era (2018-2025) | 22/33 (67%) | 13/17 (76%) | 9/16 (56%) | 0.28 |
| SMARTTOUCH STSF catheter | 25/42 (59%) | 12/16 (75%) | 13/26 (50%) | 0.19 |
| Values are presented as n (%), or mean ± standard deviation (SD).  GCV, great cardiac vein; LCC, left coronary cusp; LV, left ventricle; RF, radiofrequency; RVOT, right ventricular outflow tract. | | | | |
